# An evident asymmetrical uterus during cesarean delivery

**DOI:** 10.1002/ccr3.1802

**Published:** 2018-09-12

**Authors:** Aiko Kakigano, Shinya Matsuzaki, Mariko Jitsumori, Kazuya Mimura, Masayuki Endo, Tadashi Kimura

**Affiliations:** ^1^ Department of Obstetrics and Gynecology Osaka University Graduate School of Medicine Suita, Osaka Japan

**Keywords:** cesarean delivery, interstitial pregnancy, placenta accreta

## Abstract

If an obstetrician determines the presence of an asymmetrical uterus during cesarean delivery, the likelihood of an interstitial pregnancy complicated by placenta accreta should be considered. The figures in this article should help advance the current knowledge about a rare type of full‐term interstitial pregnancy complicated by placenta accreta.

## CASE

**Figure 1:**
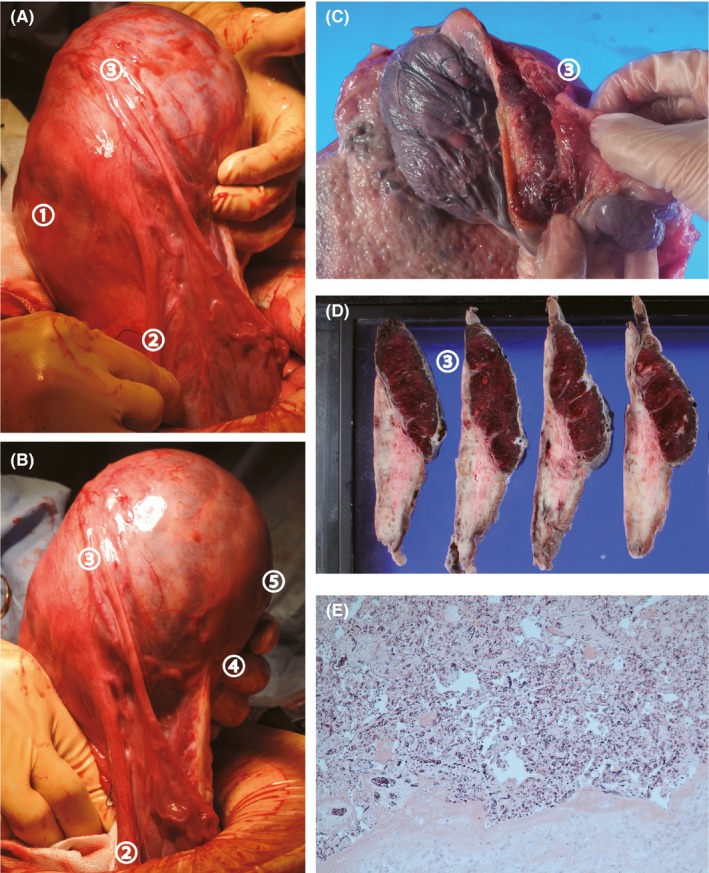
A, Left‐side view of the uterus. The uterine fundus appeared intact, with an asymmetric bulge in the interstitial part of the left fallopian tube. ① The uterine body, ② the left round ligament, and ③ the origin of the left fallopian tube. B, Posterior view of the uterus. ② The left round ligament, ③ The origin of the left fallopian tube, ④ the origin of the left ovary, and ⑤ the posterior uterine body. Based on these positional relationships, observation of the placenta, and because the distance between ③ and ④ was extended, an interstitial pregnancy complicated by placenta accreta was suspected. C, Gross findings of the surgical specimen. ③ The section around the origin of the fallopian tube. An incision in the uterus revealed that the placenta was entirely attached to the uterus with especially strong adhesion around the origin of the left fallopian tube. D, A specimen of the uterus was obtained for histopathological examination. The myometrium around the origin of the left fallopian tube was especially thin. ③ The section around the origin of the fallopian tube. E, Histopathological examination confirmed placenta accreta. In addition to the origin of the fallopian tube, the entire placenta was directly attached to the myometrium. Histopathological examination confirmed that the myometrium around the origin of the left fallopian tube was especially thin (magnification 100X)

A 33‐year‐old multiparous woman had planned a repeat cesarean delivery at gestational week 38. A healthy male infant (3148 g) was delivered but without the placenta. Intraoperative observation revealed an asymmetrical uterus. Our previous experience with a similar case suggested an interstitial pregnancy complicated by placenta accreta.^1^ The undelivered placenta was observed on the left side of the uterine fundus (Figure 1A,B); thus, a supracervical hysterectomy was performed. Gross findings of the surgical specimen revealed placental accreta near the left cornu, suggesting an interstitial pregnancy (Figure 1C,D); these findings were confirmed by histopathological examination (Figure 1E).

Interstitial pregnancies account for 3% of all tubal pregnancies.^1^ Uterine rupture usually occurs in the first trimester; thus, there have been few reports on full‐term interstitial pregnancy resulting in a live birth.^1^, ^2^ Although it is essential to check the implantation site during early pregnancy, the abnormal position of the gestational sac had not been noticed during early pregnancy by the previous doctor, and we could not detect an interstitial pregnancy after the midtrimester. Our case should prove helpful for selection of prudent management in cases of an asymmetrical uterus during cesarean delivery.

## CONFLICT OF INTEREST

None declared.

## AUTHOR CONTRIBUTIONS

AK, SM, and MJ: made substantial contributions to the conception and design of this manuscript, collected the clinical data, and drafted and revised the manuscript. ME: helped in drafting the manuscript and responded to the submission requirements. TK: conceived and generally supervised the study and gave final approval for publication of this manuscript. All authors: read and approved the final manuscript.

## INFORMED CONSENT

The patient provided written informed consent for publication of details of the diagnosis.
